# SPIDER: spatially integrated denoising via embedding regularization with single cell supervision

**DOI:** 10.1093/bioinformatics/btag430

**Published:** 2026-08-21

**Authors:** Md Istiaq Ansari, Muhtasim Noor Alif, Wei Zhang

**Affiliations:** Department of Computer Science, University of Central Florida, Orlando, FL 32816, United States; Department of Computer Science, University of Central Florida, Orlando, FL 32816, United States; Department of Computer Science, University of Central Florida, Orlando, FL 32816, United States

## Abstract

**Motivation:**

Spatial transcriptomics (ST) technologies profile gene expression while preserving tissue architecture, enabling the study of spatial cellular organization and microenvironmental interactions. However, raw ST data are heavily affected by technical noise, sparsity, and dropout events, which obscure true biological signals and hinder downstream analyses. While recent denoising methods incorporate spatial neighborhood information, they lack explicit supervision due to missing cell-type annotations in ST data. To address this challenge, we introduce SPIDER, a semi-supervised framework that leverages independently generated, annotated single-cell RNA-seq (scRNA-seq) references to guide ST denoising. SPIDER synthesizes pseudo-ST data from scRNA-seq to inject cell-type information without requiring paired measurements. The method constructs three graphs capturing spatial proximity, transcriptional similarity in real-ST data, and transcriptional structure in pseudo-ST data. Graph encoders map these representations into a shared latent space, and a domain-alignment module transfers biologically meaningful structure from pseudo-ST to real-ST embeddings. A graph-attention decoder with a zero-inflated negative binomial objective reconstructs denoised ST expression profiles.

**Results:**

We benchmark SPIDER on human dorsolateral prefrontal cortex and breast cancer datasets. SPIDER consistently enhances spatial gene expression patterns, recovers known tissue structures, and achieves superior clustering performance compared to existing approaches. Marker gene analyses demonstrate improved spatial continuity and clearer anatomical organization. By directly producing denoised expression matrices, SPIDER improves both accuracy and interpretability, providing a generalizable solution for robust ST data denoising.

**Availability and implementation:**

The source code and dataset is available at: (https://github.com/compbiolabucf/SPIDER) and archived on Zenodo (https://doi.org/10.5281/zenodo.20613921).

## 1 Introduction

Spatial transcriptomics (ST) technologies have transformed our ability to study gene expression within the original tissue architecture, enabling us to observe how cells are arranged, how they interact with their neighbors, and how different regions of a tissue function (Ståhl *et al*. 2016). New platforms such as 10x Visium, Slide-seq, Slide-seqV2, and Stereo-seq now provide large datasets with thousands of spatial spots across many tissue types (Rodriques *et al*. 2019, Maynard *et al*. 2021, Stickels *et al*. 2021, Chen *et al*. 2022). 10x Genomics Visium is one of the most widely adopted platforms for ST, offering genome-wide gene expression profiling while preserving the native spatial layout of tissue sections. It captures mRNA through spatially barcoded spots of ∼55 µm diameter, each measuring thousands of genes, and integrates histology images to connect molecular patterns with tissue morphology, spatial domains, and regional biology. Recent advances in capture chemistry have enabled higher-resolution methods such as Slide-seq and Slide-seqV2 (Rodriques *et al*. 2019, Stickels *et al*. 2021), which use barcoded beads to achieve a diameter not greater than 10 µm resolution while maintaining broad transcriptome coverage. Stereo-seq (Chen *et al*. 2022) further improves spatial precision using dense DNA nanoball arrays, enabling large-field or even whole-organ mapping at near sub-cellular scales. Imaging-based technologies such as 10x Xenium (Wu *et al*. 2021) provide true sub-cellular resolution through multiplexed fluorescence detection, typically with a reduced number of profiled genes. Together, these complementary technologies highlight the rapid evolution of ST and the growing need for computational methods that can accurately interpret gene expression within its spatial context. However, ST data often suffer from major technical challenges. Gene expression values are usually very sparse, many genes show dropout events, and overall capture rates are low (Rodriques *et al*. 2019, Du *et al*. 2023, Baul *et al*. 2024). As a result, the raw data can be noisy and incomplete, making downstream analysis such as identifying spatial domains, studying cell-cell interactions and reconstructing tissue structure much more difficult. This has created a strong need for effective computational methods that can clean, denoise, and impute ST data while keeping the true biological patterns intact.

Extensive research has focused on single-cell RNA-seq (scRNA-seq) denoising and clustering, largely because scRNA-seq technologies were developed earlier than ST technologies. Among existing tools, the well-established Seurat framework (Hao *et al*. 2021) supports a wide range of scRNA-seq analyses and can also be used to cluster or identify spatial domains in ST datasets. However, most of these methods, including newer machine learning approaches such as DCA (Eraslan *et al*. 2019), were originally designed for scRNA-seq data and therefore do not take advantage of the spatial information available in ST. To address this limitation, BayesSpace (Zhao *et al*. 2021) incorporates spatial neighbor structure through a Markov random field to encourage nearby spots to share the same cluster. More recently, several deep learning-based approaches, including DUSTED (Zhu *et al*. 2025), STAGATE (Dong and Zhang 2022), SEDR (Xu *et al*. 2024), stGRL (Lu *et al*. 2025), and DenoiseST (Cui *et al.* 2024), use graph-based techniques to capture spatial relationships and learn meaningful embeddings for downstream tasks such as clustering and denoising. These methods typically treat each spot as a node, construct a graph using spatial coordinates, and use the gene expression vector as node features. Despite these advances, some approaches do not explicitly account for dropouts and data sparsity, which are major challenges in ST analysis. DUSTED, STAGATE, SEDR, stGRL and DenoiseST address these issues and generally achieve better performance. However, most of these methods operate directly on low-dimensional latent embeddings produced by their frameworks. Although these embeddings capture useful structures, they combine information from many genes, making biological interpretation difficult. As a result, linking cluster identities back to specific genes or pathways remains challenging.

In this work, we propose SPIDER, a novel semi-supervised, spatially integrated denoising framework that leverages reference scRNA-seq data collected from the same tissue type as the ST dataset. The scRNA-seq reference provides per-cell expression profiles and cell-type annotations derived from clustering and marker-gene analysis (Hao *et al*. 2021). By pooling cells of known types into pseudo spots and using their cell-type compositions as supervision, the model learns biologically meaningful mixtures in its latent space. This supervision helps infer cell-type composition in unannotated ST data and improves denoised expression profiles. A zero-inflated negative binomial loss captures key statistical properties of ST data, including overdispersion and dropouts (Risso *et al*. 2018). We evaluate SPIDER on multiple datasets against several baseline methods. Unlike many existing approaches, downstream analyses are performed on the final denoised expression matrix rather than low-dimensional latent embeddings. This design preserves gene-level interpretability, allowing biological signals to be traced directly to individual genes and supporting clearer biological validation.

## 2 Methods

### 2.1 Problem formulation

Let a ST dataset be represented by a raw count matrix $X_{\text{st}}\in R^{N_{s}\times G}$, where $N_{s}$ denotes the number of spots and each spot is described by a gene expression vector of length *G*. Each spot *i* further has an associated 2D spatial coordinate $c_{i}\in R^{2}$. Our goal is to recover, for every spot *i*, a denoised gene expression vector that mitigates various sources of technical and biological noise. To guide the denoising process, we make use of a scRNA-seq dataset $X_{\text{sc}}\in R^{N_{\text{sc}}\times G}$, where $N_{\text{sc}}$ is the number of single cells, profiled from the same tissue type as the ST data. From this scRNA-seq reference, we construct a pool of pseudo spots (i.e., pseudo-ST), $X_{\text{psd}}\in R^{N_{p}\times G}$, and employ embedding alignment between the ST spots and these pseudo spots to inform the denoiser. In summary, given the raw ST data $\left(X_{\text{st}},{\left\{c_{i}\right\}}_{i=1}^{N_{s}}\right)$ and the reference scRNA-seq data $X_{\text{sc}}$, our goal is to generate a denoised ST expression matrix ${\hat{X}}_{\text{st}}\in R^{N_{s}\times G}$.

### 2.2 Overview of the SPIDER framework

**Figure 1:**
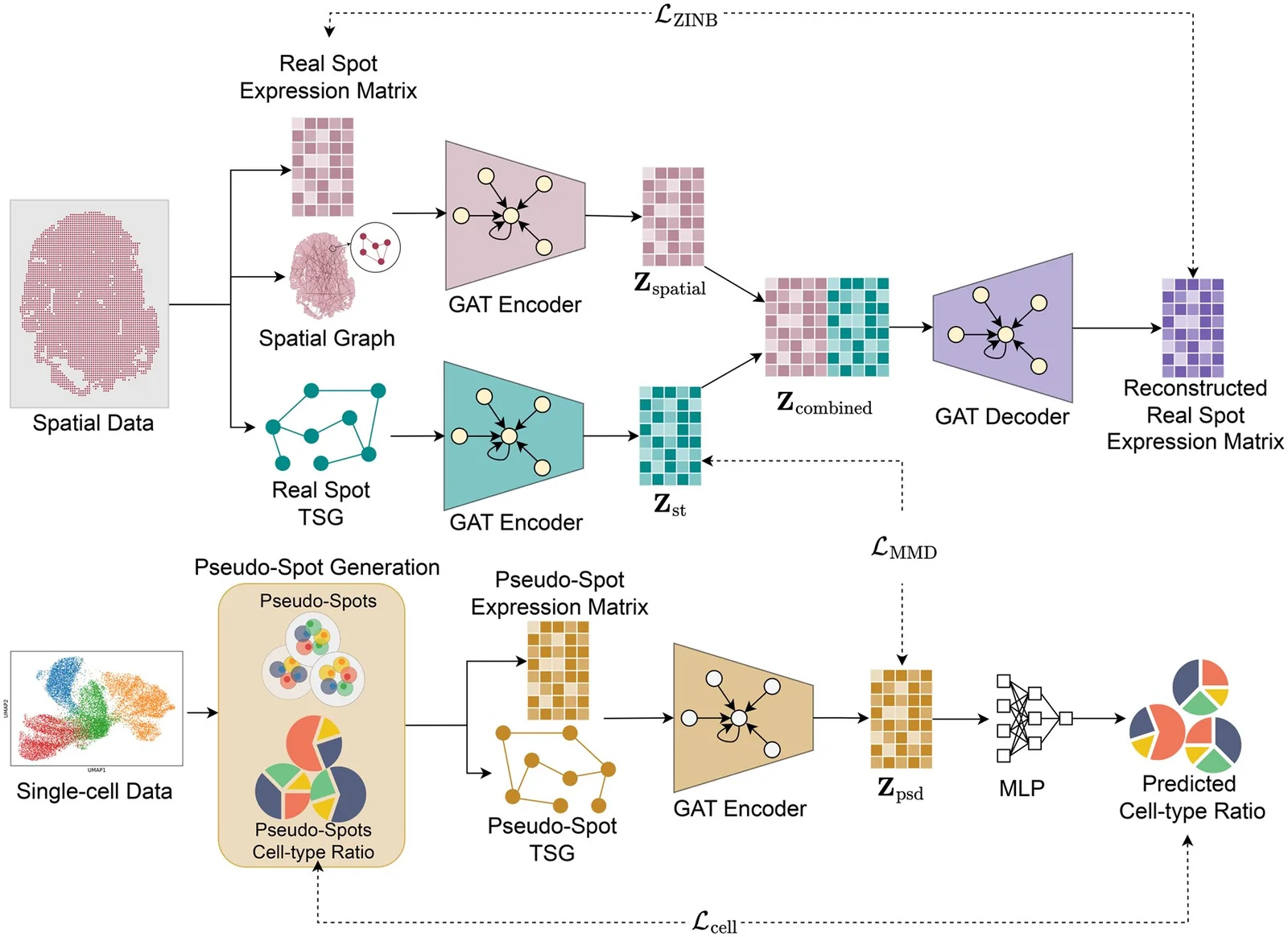
Overview of SPIDER framework. SPIDER integrates ST data with reference scRNA-seq information by jointly leveraging spatial structure, transcriptional similarity, and pseudo-ST supervision. The framework first constructs three graphs: a spatial neighborhood graph derived from spot coordinates, and two transcriptional similarity graphs (TSGs) built from real-ST and pseudo-ST generated from scRNA-seq data from the same tissue type. A tri-branch graph attention encoder then learns low-dimensional latent representations from these graphs. The pseudo-ST branch incorporates supervision by optimizing cell-type ratio prediction, while an embedding regularization term aligns the real-ST and pseudo-ST embeddings to transfer cell-type structure from the reference domain. A unified graph attention decoder reconstructs gene expression using a zero-inflated negative binomial likelihood. The resulting denoised expression matrix can be used for downstream analyses, including spatial domain identification, marker gene pattern recovery, and improved clustering performance.

Our proposed framework, SPIDER, denoises ST data by integrating spatial neighborhood structure with transcriptional similarity and by transferring cell-type–aware supervision from single-cell references through pseudo-ST data. As illustrated in Fig. 1, the pipeline has three stages: (i) pseudo-ST generation, (ii) graph construction, and (iii) model training. We first apply log-normalization to raw ST and scRNA-seq count matrices to improve comparability across samples and reduce skewness. From the preprocessed scRNA-seq data, we then generate pseudo-ST spots by randomly mixing cells from different cell types, yielding synthetic spots with known cell-type composition ratios that provide supervision during training.

We next construct three graphs: two from real-ST data and one from pseudo-ST data. For real ST, we build a spatial graph from spot coordinates to encode Euclidean proximity, and a transcriptional similarity graph (TSG) from expression profiles using k-nearest neighbors with Euclidean distance. We construct an analogous TSG for pseudo-ST data. Together, these graphs capture complementary spatial and molecular relationships and enable propagation of both biological and spatial information across nodes.

The graphs are then fed into a dual-domain graph attention autoencoder with one encoder for the real-ST spatial graph and two separate encoders for the real-ST and pseudo-ST TSGs. Because pseudo-ST spots have known cell-type compositions, the pseudo-ST TSG encoder is trained with a cross-entropy loss to predict composition ratios, encouraging biologically meaningful latent representations. In contrast, real-ST spots lack such annotations. Moreover, pseudo-ST spots are generated by random aggregation of single cells, whereas real-ST spots reflect spatially organized cellular neighborhoods, making spot-level alignment between the two domains ill-defined. To bridge this gap, we use Maximum Mean Discrepancy (MMD) (Gretton *et al*. 2012), which aligns the distributions of latent embeddings rather than individual samples. This allows transfer of cell-type–aware structure as a soft biological prior while accommodating differences in local composition imposed by spatial organization.

The latent embedding from the spatial encoder is combined with the aligned latent embedding from the real-ST TSG encoder and passed to a graph attention decoder. The decoder estimates the mean, inverse dispersion, and zero-inflation probability of a Zero-Inflated Negative Binomial (ZINB) distribution, which defines the reconstruction loss and produces denoised expression profiles for real-ST spots. By combining pseudo-ST supervision with latent-space alignment, SPIDER learns biologically meaningful representations that enable more accurate reconstruction of ST gene expression. The following sections describe each stage in detail, and Table 1 summarizes the notation used throughout the manuscript.

**Table 1:** Summary of notations used in the SPIDER framework.

Symbol	Description
$N_{s}$, $N_{p}$, *G*, *C*	Number of real-ST spots, pseudo spots, genes per spot, and cell-types
$X_{\text{st}}\in R^{N_{s}\times G}$	Expression matrix of real-ST data
$X_{\text{sc}}\in R^{N_{\text{sc}}\times G}$	Expression matrix of scRNA-seq reference
$X_{\text{psd}}\in R^{N_{p}\times G}$	Simulated pseudo-ST expression matrix
$c_{i}\in R^{2}$	Spatial coordinates of spot *i*
${\hat{X}}_{\text{st}}\in R^{N_{s}\times G}$	Denoised ST gene expression matrix
$y_{j}$ , ${\hat{y}}_{j}\in \Delta^{C-1}$	Ground-truth and predicted cell-type composition vectors of pseudo-ST *j*
$G_{\text{sp}}=(V_{\text{real}},E_{\text{sp}})$	Spatial graph constructed from real-ST data
$G_{\text{expr}}^{\text{st}}=(V_{\text{real}},E_{\text{expr}}^{\text{st}})$	Transcriptional similarity graph (TSG) of real-ST data
$G_{\text{expr}}^{\text{psd}}=(V_{\text{psd}},E_{\text{expr}}^{\text{psd}})$	Transcriptional similarity graph (TSG) of pseudo-ST data
$Z_{\text{spatial}}$ , $Z_{\text{st}}$ , $Z_{\text{psd}}$	Latent embeddings from spatial encoder, real-ST TSG, pseudo-ST TSG
μ,θ,π	Parameters of decoder: mean, inverse dispersion, zero-inflation probability
$L_{\text{ZINB}}$ , $L_{\text{cell}}$ , $L_{\text{MMD}}$ , L	Reconstruction loss, cell-type ratio loss, alignment loss, and total loss
$\lambda_{\text{rec}},\lambda_{\text{cell}},\lambda_{\text{mmd}}$	Weight coefficients for respective loss terms

### 2.3 Pseudo-ST simulation

To provide explicit supervision for cell-type composition, we generate pseudo-ST spots from scRNA-seq data with known cell-type annotations. The goal is to mimic the mixture complexity of real-ST spots while avoiding biases from imbalanced cell-type frequencies in the reference.

For each pseudo spot, we first sample the number of distinct cell types, $k∼\text{Uniform}\{1,\ldots ,C_{\max}\},$ where $C_{\max}$ is a user-defined upper bound chosen according to the expected complexity of the target platform and tissue. We then select *k* distinct cell types uniformly from the full set of *C* annotated types, ensuring comparable sampling probability for rare and abundant cell types.

Next, we sample the total number of contributing cells for pseudo spot *j*, $M_{j}∼\text{Uniform}(M_{\min},M_{\max}),$ where $M_{\min}$ and $M_{\max}$ specify a plausible range of cells per spot. Individual cells are randomly drawn from the selected cell types with equal probability within each type, allowing cell-type proportions to vary across pseudo spots.

The expression profile of each pseudo spot is obtained by summing the raw gene counts of its sampled cells. Because cell-type labels are known in the scRNA-seq reference, the resulting composition can be computed exactly and used as supervision. We denote the pseudo-ST expression matrix by $X_{\text{psd}}\in R^{N_{p}\times G}$, where $N_{p}$ is the number of pseudo spots and *G* is the number of genes. The composition of pseudo spot *j* is denoted by $y_{j}\in \Delta^{C-1}$, where *C* is the total number of cell types.

### 2.4 Graph construction

To encode the topological relationships among spots in both gene expression and spatial domains, we construct three graphs from the real-ST data $X_{\text{st}}$ and the pseudo-ST data $X_{\text{psd}}$. Let $V_{\text{real}}$ and $V_{\text{psd}}$ denote the node sets corresponding to real-ST and pseudo-ST, respectively, where $|V_{\text{real}}|=N_{s}$ and $|V_{\text{psd}}|=N_{p}$. First, we build a spatial graph $G_{\text{sp}}=(V_{\text{real}},E_{\text{sp}})$ that captures the spatial neighborhood structure of the real-ST, where edges are defined based on Euclidean distances between spot coordinates in the tissue. Second, we construct a TSG on the real-ST data, $G_{\text{expr}}^{\text{st}}=(V_{\text{real}},E_{\text{expr}}^{\text{st}})$, where edges are formed based on pairwise expression similarity in $X_{\text{st}}$ and the gene expression vectors are the node feature. Third, we build an analogous TSG on the pseudo-ST expressions $X_{\text{psd}}$, denoted by $G_{\text{expr}}^{\text{psd}}=(V_{\text{psd}},E_{\text{expr}}^{\text{psd}})$, using the same construction procedure. These graphs are subsequently used in the graph encoders and decoders within our framework to facilitate structured information propagation.

### 2.4 Modeling and training

We use a dual-domain graph attention (GAT) (Veličković *et al*. 2017) autoencoder that jointly integrates spatial structure and gene-expression structure while transferring supervision from pseudo-ST data created from the reference scRNA-seq data to real-ST spots. Unlike prior denoisers that rely solely on ST count matrices, our framework injects single-cell–derived supervision via pseudo-ST and enforces alignment between the latent embeddings learned from pseudo-ST and real-ST domains.

#### 2.4.1 Graph attention autoencoder

We employ three GAT-based encoders to capture spatial and transcriptional relationships between the real-ST and pseudo-ST, together with a GAT-based decoder that reconstructs denoised gene-expression profiles for the real-ST.

*Graph Attention*. Given a graph G=(V,E) and node features $X\in R^{N\times F}$, a single-head GAT layer computes: $\text{GAT}(X,G)=H^{\prime }\in R^{N\times F^{\prime }},$ where


$$\begin{array}{c} H_{i}^{\prime }=\sigma \left(\sum_{j:(i,j)\in E}\frac{e^{\ell_{ij}}}{\sum_{k:(i,k)\in E}e^{\ell_{ik}}}X_{j}W\right),\\ \ell_{ij}=\mathrm{LeakyReLU}\left(a^{⊤}[X_{i}W\|X_{j}W]\right),\;i=1,\ldots ,N. \end{array}$$

Here, *N* denotes the number of nodes, *F* and $F^{\prime }$ are the input and output feature dimensions, $W\in R^{F\times F^{\prime }}$ is a learnable weight matrix, $a\in R^{2F^{\prime }}$ is a learnable attention vector, [·‖·] denotes vector concatenation, and σ(·) is a nonlinear activation function.

*Encoder(s)*. In our framework, each encoder stacks two GAT layers and produces node embeddings: $Z={\text{GAT}}^{\left(2\right)}\left(\text{ELU}({\text{GAT}}^{\left(1\right)}(X,G))\right),$ where G is the corresponding graph, ELU(.) is the non-linear activation function, and Z is the resulting embedding. The spatial encoder operates on the spatial graph $G_{\text{sp}}=(V_{\text{real}},E_{\text{sp}})$ and yields spatial embeddings $Z_{\text{spatial}}$. The other two encoders are applied to the TSGs of the real-ST data and pseudo-ST data, $G_{\text{expr}}^{\text{st}}=(V_{\text{real}},E_{\text{expr}}^{\text{st}})$ and $G_{\text{expr}}^{\text{psd}}=(V_{\text{psd}},E_{\text{expr}}^{\text{psd}})$, producing embeddings $Z_{\text{st}}$ and $Z_{\text{psd}}$, respectively.

*Decoder*. The decoder follows the same GAT-based design and integrates spatial and transcriptional information to reconstruct denoised expression for the real-ST. The embeddings $Z_{\text{st}}$ and $Z_{\text{spatial}}$ are first concatenated and passed through a GAT layer followed by an ELU activation to form a unified representation:


$$Z_{\text{combined}}=\text{ELU}\left({\text{GAT}}^{\left(\text{combined}\right)}([Z_{\text{st}}||Z_{\text{spatial}}],G_{\text{expr}}^{\text{st}})\right).$$

The combined embedding $Z_{\text{combined}}$ is fed into three parallel GAT layers that output the ZINB parameters μ, θ, and π:


$$r=\phi_{r}\left({\text{GAT}}^{\left(r\right)}(Z_{\text{combined}},G_{\text{expr}}^{\text{st}})\right),\;r\in \{\mu ,\theta ,\pi \},$$

Where $\phi_{\mu }=\text{exponential},\phi_{\theta }=\text{softplus}$ and $\phi_{\pi }=\text{sigmoid}$, ensuring μ>0, θ>0, and 0<π<1 for all spots and genes.

#### 2.4.2 Training

To train our framework, we impose three main constraints: one for gene expression reconstruction, one for pseudo-ST supervision, and one for domain alignment. Together, these objectives encourage the model to learn cell-type–informative representations from pseudo-ST, transfer this structure to the real-ST domain, and faithfully recover denoised expression profiles. This joint optimization ensures that spatial, transcriptional, and cross-domain signals are coherently integrated during training.

##### 2.4.3 Gene expression reconstruction

To faithfully recover denoised gene-expression profiles while respecting the discrete and sparse nature of ST data, we model the decoder output using a ZINB likelihood. Recall that the decoder takes the combined embedding $Z_{\text{combined}}$ and uses three parallel GAT-based heads to predict the ZINB parameters (μ,θ,π) for each spot and gene. The reconstruction loss is defined as $L_{\text{ZINB}}(x;\mu ,\theta ,\pi )=-\text{log}\;P(X=x|\mu ,\theta ,\pi )$, where μ denotes the mean expression, θ the inverse dispersion, π the zero-inflation probability, and *x* the raw count matrix of the ST data. This likelihood is well suited to the statistical characteristics of ST count data, particularly high sparsity, overdispersion, and dropout, and therefore supports biologically meaningful and statistically robust reconstruction.

##### 2.4.4 Pseudo-ST supervision

To transfer high-resolution cell-type information from scRNA-seq to the spatial domain, we use the known cell-type composition of each pseudo spot as a supervision signal. The pseudo-ST embedding $Z_{\text{psd}}$ is passed through a multilayer perceptron (MLP) followed by a softmax layer to predict the cell-type composition vector, $\hat{y}=\text{softmax}\left(\text{MLP}(Z_{\text{psd}})\right)$. We train this branch using a categorical cross-entropy loss, $L_{\text{cell}}=-\frac{1}{N_{p}}\sum_{j=1}^{N_{p}}\sum_{c=1}^{C}y_{jc}\;\text{log}({\hat{y}}_{jc})$, where $y_{jc}$ is the ground-truth proportion of cell type *c* in pseudo spot *j*. This supervision encourages the encoder to learn cell-type–informative, high-resolution representations from the scRNA-seq–derived pseudo-ST.

##### 2.4.5 Domain alignment

To transfer cell-type–aware structure from the supervised pseudo-ST domain to the unsupervised real-ST domain, we align their latent representations. The features learned from the pseudo-ST encoder are used to guide the real-ST encoder so that the resulting embeddings also capture meaningful gene-expression structure. Concretely, we align the embeddings $Z_{\text{st}}$ and $Z_{\text{psd}}$ using a MMD loss with a multi-bandwidth RBF kernel: $L_{\text{MMD}}=\|\phi (Z_{\text{st}})-\phi (Z_{\text{psd}}){\|}_{H}^{2},$ where ϕ(·) denotes the feature map into the reproducing kernel Hilbert space H. The aligned real-ST embeddings, enriched by supervision from the pseudo-ST domain, are then combined with the spatial embeddings and passed to the decoder for expression reconstruction.

##### 2.4.6 Overall objective function

The total loss function jointly optimizes denoising, domain alignment, and pseudo-ST supervision:


$$L=\lambda_{\text{rec}}L_{\text{ZINB}}+\lambda_{\text{mmd}}L_{\text{MMD}}+\lambda_{\text{cell}}L_{\text{cell}},$$

Where $\lambda_{\text{rec}}$, $\lambda_{\text{mmd}}$, and $\lambda_{\text{cell}}$ are weighting coefficients that balance the contribution of each term. Model parameters are optimized using the Adam optimizer with gradient clipping, and hyperparameters are tuned using the Optuna (Akiba *et al*. 2019) framework to achieve the best denoising performance. In particular, the loss weights require careful adjustment because different scRNA-seq references may contribute unevenly to the ST dataset, and the pseudo-ST generation process does not guarantee that the resulting distribution of cell types or gene expression perfectly matches real-ST data. Tuning these weights is therefore essential to control how strongly information from the pseudo-ST domain and the alignment objective influences the learned representations and, ultimately, the denoising of the real-ST data.

## 3 Experiments and results

### 3.1 Datasets and preprocessing

#### 3.1.1 Datasets and baselines

To evaluate the performance of SPIDER, we compare it with the state-of-the-art ST denoising and representation learning methods, DUSTED (Zhu *et al*. 2025), STAGATE (Dong and Zhang 2022), and SEDR (Xu *et al*. 2024), stGRL (Lu *et al*. 2025), DenoiseST (Cui *et al.* 2024) and also include the raw ST data as a baseline. Because our method uses single-cell information as guidance, each ST dataset is paired with its corresponding scRNA-seq or snRNA-seq (single-nucleus RNA-seq) reference. For human brain data, we use the DLPFC Visium dataset (Maynard *et al*. 2021), which contains 12 tissue sections (∼4000 spots per section, ∼33k genes). Each tissue section has annotated spatial domains. The matched DLPFC snRNA-seq dataset (Russell *et al*. 2024) includes ∼17k nuclei and ∼36k genes. We also evaluate on the CID44971 breast cancer dataset (Wu *et al*. 2021), which contains 1162 Visium spots (∼20k genes) and pathologist-annotated labels spanning seven microenvironmental tissue regions, along with an accompanying scRNA-seq dataset of 7,986 cells (∼30k genes).

#### 3.1.2 Preprocessing

For each dataset, we retain genes shared between the ST and single-cell matrices, remove out-of-tissue spots, normalize counts, and apply log transformation. The normalized ST matrix is used as the input to the model and the raw count matrix is used in the loss function to calculate loss. We simulate pseudo-ST data from the single-cell references following (Li and Luo 2024). For each synthetic spot, we sample a small randomized number of cell types and a realistic but randomized total number of cells, which are then stochastically drawn from the selected cell types and aggregated. This produces heterogeneous yet biologically plausible mixtures reflecting typical ST spot composition.

### 3.2 SPIDER generates denoised ST expression for accurate clustering

We evaluate SPIDER on its ability to recover biologically meaningful spatial domains, comparing it with the raw data and representative ST denoising and representation learning methods on two benchmark datasets. To ensure a fair comparison, all methods are evaluated using the same clustering pipeline: each denoised expression matrix is normalized, reduced by PCA using the same number of components, and clustered with mclust (Scrucca *et al*. 2016). This setup ensures that performance differences reflect the quality of the denoised expression rather than method-specific latent embeddings. Although baseline methods typically cluster latent embeddings, we cluster their denoised expression matrices as we focus on the denoise ST matrix. Clustering performance is assessed using Normalized Mutual Information (NMI), Adjusted Rand Index (ARI), and Homogeneity Score (HS).

The results are reported in Table 2. SPIDER consistently achieves the highest overall clustering accuracy on the DLPFC and breast cancer (CID44971) datasets, both of which provide ground-truth annotations for spatial domain assignments. Together, these results demonstrate SPIDER’s robust spatial domain recovery across diverse tissues and measurement technologies.

**Table 2:** *Clustering performance of SPIDER* and baseline methods across two ST datasets, DLPFC section 151673 and CID44971 breast cancer dataset.

Dataset	Model	NMI	ARI	HS
DLPFC (151673)	Raw	0.4568	0.2956	0.4522
	DUSTED	0.6787	0.5212	0.6693
	STAGATE	0.6434	0.4804	0.6301
	SEDR	0.4549	0.2896	0.4564
	stGRL	0.6140	0.5067	0.6012
	DenoiseST	0.5194	0.3346	0.5224
	SPIDER	**0.7206**	**0.5853**	**0.7057**
CID44971	Raw	0.5620	0.4487	0.5314
	DUSTED	0.6916	0.6231	0.6674
	STAGATE	0.6663	0.5798	0.6288
	SEDR	0.6731	0.5667	0.6422
	stGRL	0.6310	0.5463	0.5990
	DenoiseST	0.6017	0.5570	0.5788
	SPIDER	**0.6981**	**0.6283**	**0.6757**

Normalized Mutual Information (NMI), Adjusted Rand Index (ARI), and Homogeneity Score (HS) were computed using mclust on PCA-reduced denoised expression matrices produced by each method. Bold values represent the best performing method.

**Figure 2:**
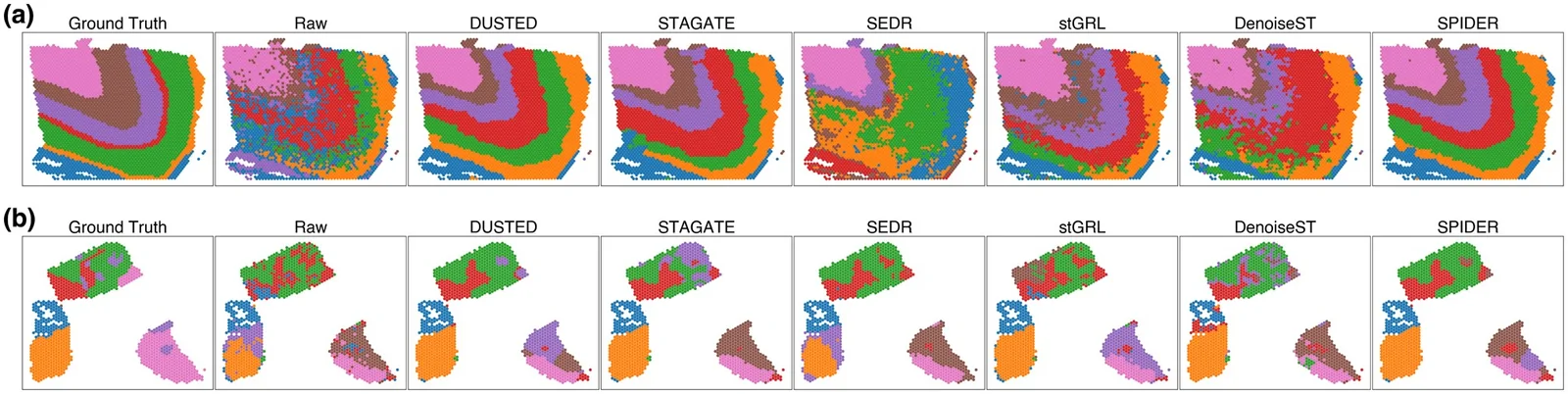
SPIDER improves domain recovery across different datasets. Comparison between baseline methods and SPIDER is shown in the figure for (a) DLPFC section 151673 for recovering different layers of the tissue and (b) CID44971 breast cancer dataset for identifying pathologist-derived tissue region annotations.

On the DLPFC dataset, SPIDER produces spatial domains that closely align with the anatomical cortical layers (L1–L6 and white matter). As shown in Fig. 2a, the raw data, SEDR, stGRL and DenoiseST yield fragmented and spatially inconsistent domains, often failing to delineate clear laminar boundaries due to noise and local discontinuities. DUSTED and STAGATE partially recover the laminar organization but still exhibit mixing between adjacent layers and irregular boundary delineation. In contrast, SPIDER generates sharper and more continuous laminar structures with minimal fragmentation and well-defined transitions between consecutive layers.

**Figure 3:**
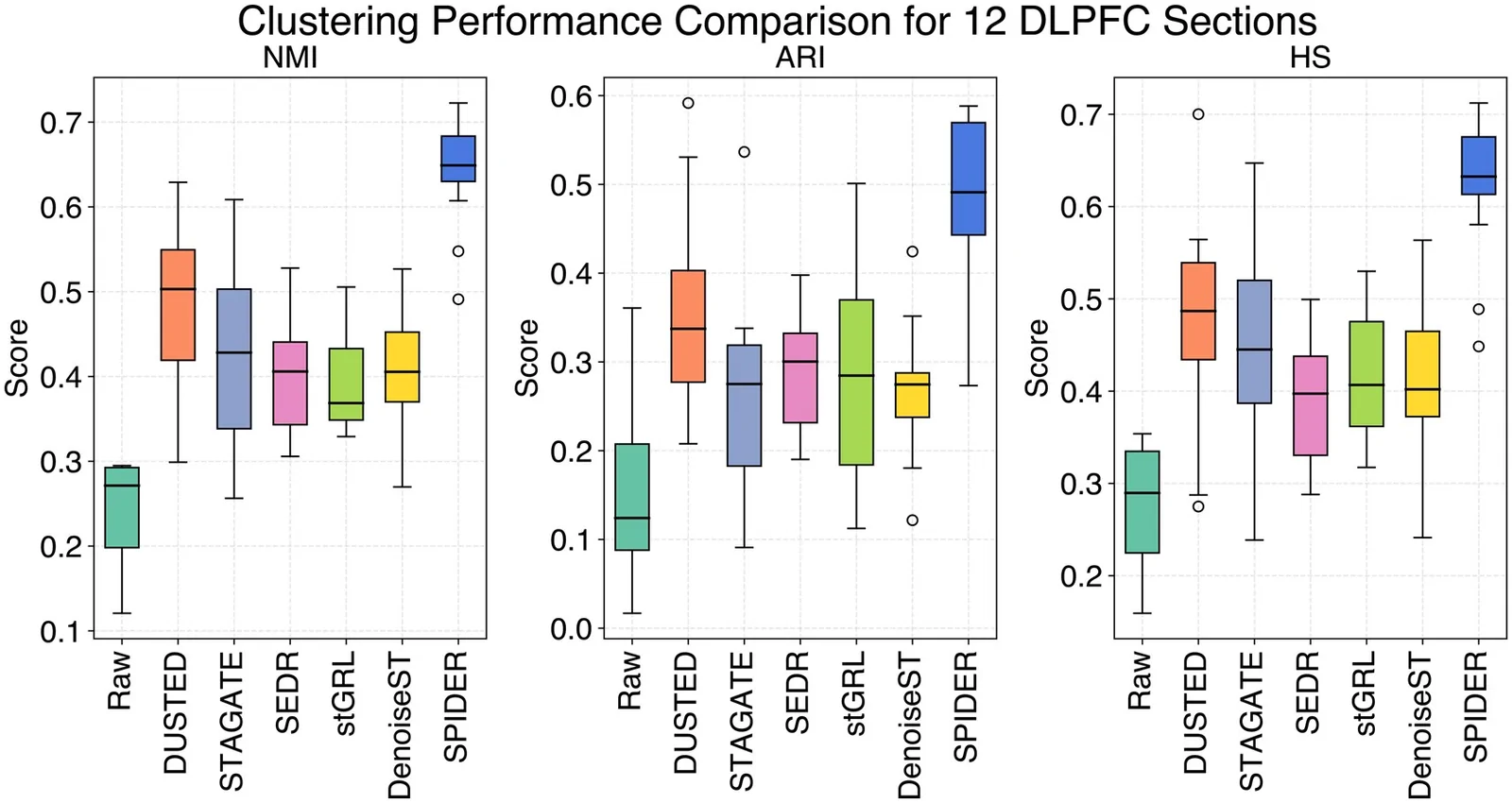
SPIDER shows consistent performance across 12 sections of DLPFC dataset. Figure shows the boxplot for clustering performance metrics NMI, ARI and HS for 12 sections of the DLPFC dataset for baseline methods and SPIDER.

These improvements suggest that SPIDER effectively reduces noise while preserving layer-specific biological variation. Quantitatively, SPIDER achieves the best performance in terms of NMI (0.7206), ARI (0.5853) and HS (0.7057), outperforming all baselines by a substantial margin. This demonstrates the model’s ability to capture both global and local structure in the denoised gene expression matrix. Across all 12 DLPFC sections, SPIDER consistently outperforms baseline methods in average NMI, ARI and HS (Fig. 3), highlighting its robustness to biological variability and differences in spatial organization across tissue slices. Importantly, the stability of SPIDER’s performance across multiple sections indicates that it does not rely on dataset-specific artifacts and generalizes well across independent samples.

For the CID44971 breast cancer dataset, SPIDER continues to demonstrate strong performance. As shown in Fig. 2b, the raw data, SEDR, stGRL and DenoiseST fail to clearly separate malignant, stromal, and immune-infiltrating regions, while STAGATE and DUSTED partially delineate region-specific boundaries but still show irregularities and mixing between subregions. In contrast, SPIDER produces the most coherent and biologically consistent clustering map, accurately capturing tumor cores, invasive margins, and distinct stromal compartments. The clustering metrics reflect this trend: SPIDER achieves the highest NMI (0.6981), ARI (0.6283) and HS (0.6757), demonstrating superior robustness even in highly heterogeneous microenvironments.

Overall, SPIDER demonstrates superior clustering performance across datasets and metrics, highlighting its ability to denoise ST data in a way that preserves biologically meaningful structures and enhances spatial domain identification. Further evidence of SPIDER’s performance is shown via the UMAP and PAGA analysis in the supplementary document.

### 3.3 SPIDER enhances marker gene expression

**Figure 4:**
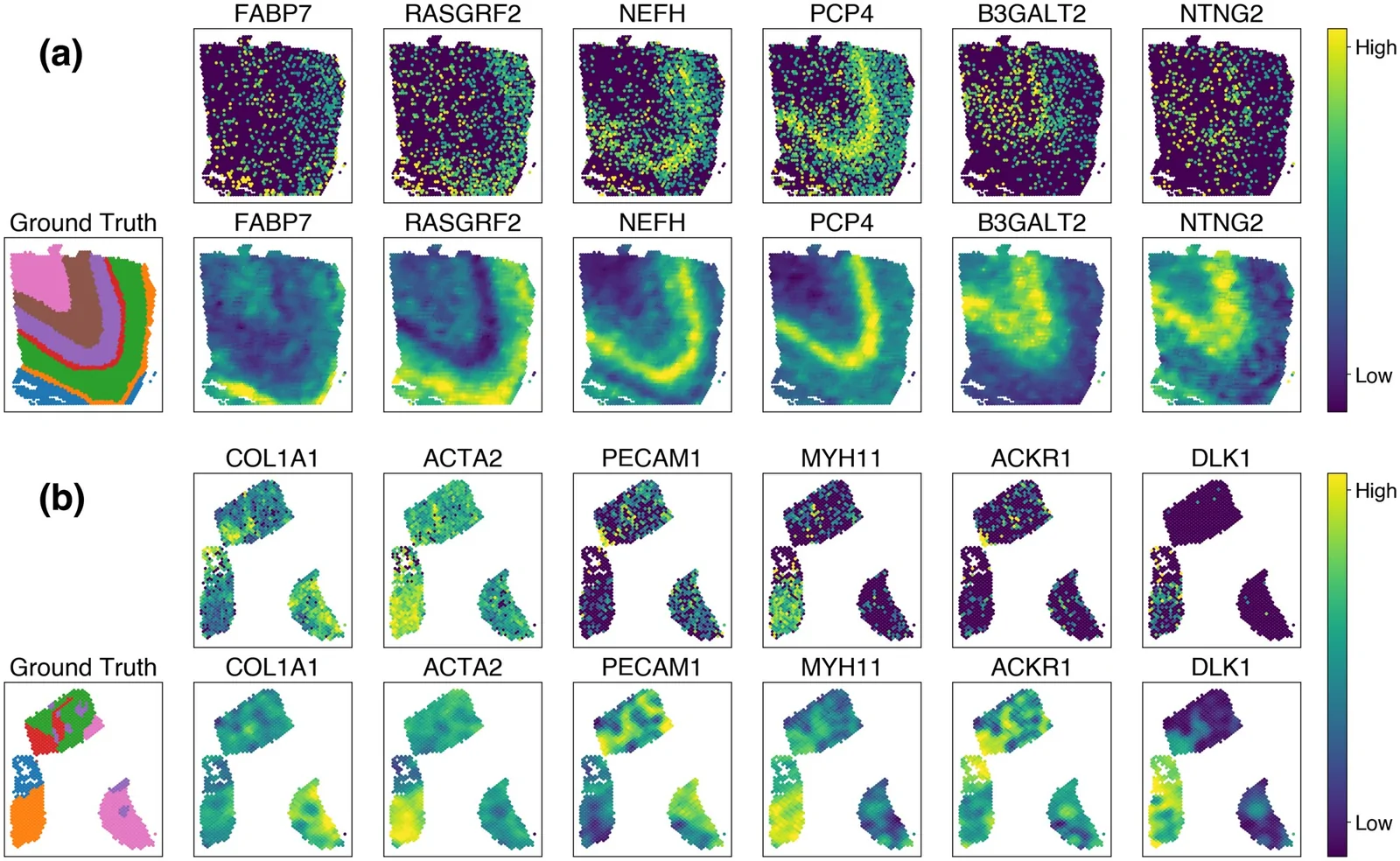
SPIDER enhances the marker gene expression patterns. Six marker genes are presented before and after denoising for (a) the DLPFC section 151673 and (b) the CID44971 breast cancer dataset. The first row displays the raw expression, and the second row shows the expression denoised by SPIDER.

SPIDER substantially enhances the visibility and spatial coherence of key marker genes relative to the raw data. In the DLPFC section 151673, Fig. 4a shows six well-established marker genes (Strehler 2015, Maynard *et al*. 2021). In the raw expression maps (top row), marker signals are noisy, with low signal-to-noise ratio and blurred separation between cortical layers. After denoising with SPIDER, these patterns become markedly sharper: expression is more spatially localized and layer boundaries are more distinct, indicating improved marker-gene recovery while preserving genuine biological heterogeneity.

A similar effect is observed in the CID44971 breast cancer dataset, where SPIDER enhances markers associated with distinct tissue compartments and immune infiltration patterns (Wu *et al*. 2021). As shown in Fig. 4b, the denoised data restore clear spatial localization of tumor-associated, stromal, and immune-cell markers, facilitating visual identification of microenvironmental niches. In several cases, SPIDER also reveals subtle gradients and compartment-specific enrichment that are barely detectable in the raw data. These results show that SPIDER improves not only clustering performance but also gene-level interpretability, which is critical for downstream biological analysis.

For quantitative evaluation, we define the within-domain average correlation as the mean pairwise Pearson correlation between gene-expression vectors of all spot pairs within the same annotated spatial domain, and the out-domain average correlation as the corresponding mean over spot pairs from different domains. Their difference measures the enrichment of transcriptional similarity within spatial domains. On DLPFC, the raw data exhibit only modest separation between within- and out-domain correlations (0.437 vs. 0.380; difference = 0.056), indicating limited domain-specific coherence due to technical noise. After denoising with SPIDER, both correlations increase substantially (within-domain = 0.967, out-domain = 0.859), and the separation nearly doubles (difference = 0.108), demonstrating stronger domain-specific coherence while preserving inter-domain distinctions. In contrast, the raw CID44971 data show almost no separation (0.439 vs. 0.437; difference = 0.002), consistent with a highly heterogeneous tumor microenvironment. After denoising, overall correlations increase (0.683 vs. 0.650) while the separation remains small (difference = 0.033), indicating effective noise reduction without artificially imposing strong domain-level homogeneity.

## 4 Conclusion

SPIDER is a semi-supervised ST denoising framework that integrates spatial structure with supervision from matched scRNA-seq data. By combining spatial neighborhoods, transcriptional similarity, and pseudo-ST data derived from scRNA-seq, it generates denoised gene-expression profiles that improve downstream analyses while transferring cell-type information to real-ST spots without disrupting their spatial context. The importance of the single-cell integration is shown in the ablation study in the supplementary document.

Across diverse datasets, SPIDER improves spatial pattern recovery, domain separability, and marker-gene expression, and supports downstream tasks including differential expression analysis, visualization, and biological validation. Because these gains are reflected directly in the denoised expression matrix, the framework preserves gene-level interpretability and ties performance improvements to biologically meaningful expression changes rather than abstract latent features. Its reconstruction-and-alignment design also makes it extensible to related tasks, such as scRNA-seq denoising with spatial priors and imputation of missing or unmeasured genes in ST data.

Despite these strengths, SPIDER depends on high-quality matched single-cell references, which may still differ from ST data in their technical biases. SPIDER is also computationally slower than the other methods as shown in the supplementary document. Future work should therefore focus on learning more transferable representations and scaling the framework to increasingly large, high-resolution, and multi-omic ST datasets.

Overall, SPIDER provides a biologically grounded approach to ST denoising that bridges spatial context and single-cell transcriptional information, improving data quality, strengthening downstream analyses, and advancing the study of tissue spatial organization.

### Author contributions

Md Istiaq Ansari (Conceptualization [equal], Data curation [equal], Formal analysis [lead], Methodology [equal], Software [lead], Validation [lead], Writing—original draft [lead]), Muhtasim Noor Alif (Investigation [supporting], Methodology [supporting], Writing—original draft [supporting]), and Wei Zhang (Conceptualization [equal], Funding acquisition [lead], Investigation [equal], Methodology [equal], Resources [equal], Supervision [lead], Writing—original draft [supporting])

## Supplementary Material

btag430_Supplementary_Data

## Data Availability

The data underlying this article are available in Zenodo at DOI: 10.5281/zenodo.20613921. The source code used in this study is available in the SPIDER GitHub repository and is also archived in Zenodo at the same DOI.
