# Supplementary material for: SPIDER: spatially integrated denoising via embedding regularization with single cell supervision
Source: Bioinformatics. 2026 Aug 21;42(Suppl 2):btag430. doi: 10.1093/bioinformatics/btag430 (PMC13501285; doi:10.1093/bioinformatics/btag430)
Supplement: Supplementary file 1 [file btag430_supplementary_data.pdf]

Supplementary Material

SPIDER: Spatially Integrated Denoising via Embedding Regularization with Single Cell Supervision

Md Istiaq Ansari[1], Alif Muhtasim[1], Wei Zhang[1]

[1]Department of Computer Science, University of Central Florida, Orlando, FL 32816
Correspondence: wzhang.cs@ucf.edu

Contents

S1 UMAP and PAGA visualization 2

S2 Ablation Study 2

S3 Runtime Analysis 3

S1 UMAP and PAGA visualization

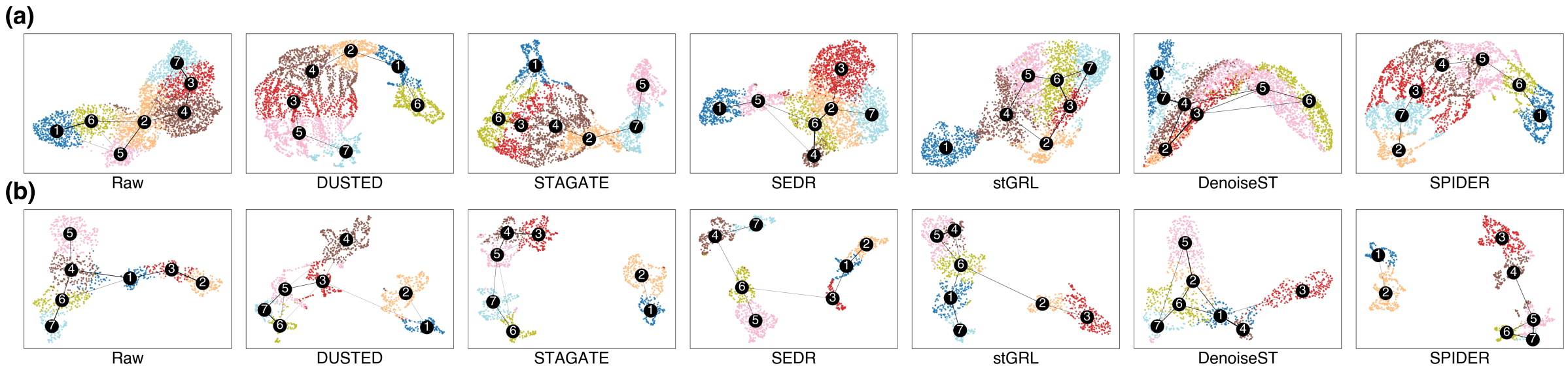

Figure S1: **UMAP and PAGA visualizations reveal SPIDER improves global topology in clustering.** Figure shows the UMAP clusters and PAGA trajectory for the **(a)** DLPFC dataset and **(b)** CID44971 breast cancer dataset.

We also visualize the UMAP [1] embeddings of the denoised expression matrices for each method, including the raw expression (Fig. S1). In addition, we overlay the Partition-based Graph Abstraction (PAGA) [2] trajectories on the UMAP plots to illustrate how clusters are connected within the underlying data manifold. PAGA reveals both the cluster identities and the transitions between clusters. An orderly, linear connectivity in the PAGA graph indicates that the method preserves continuous biological relationships while still identifying discrete spatial domains. On the DLPFC dataset, DUSTED and SPIDER produce the strongest clustering performance, each yielding a largely linear PAGA trajectory, whereas the other methods exhibit loops and branching structures in the inferred connectivity graph (Fig. S1(a)). For the breast cancer dataset, however, DUSTED loses consistency and forms multiple loops, and STAGATE shows similar instability. SEDR produces a more linear trajectory on the breast cancer data but introduces large jumps between nodes 3–6 instead of separating the two clusters like SPIDER does, suggesting a loss of global tissue structure. stGRL does similar as SPIDER for the breast cancer dataset but fails on DLPFC dataset by producing loops. DenoiseST produces multiple loops for both datasets which is not ideal in the paga plot. In contrast, SPIDER shows a coherent connectivity pattern, with only a small jump and a single loop.

S2 Ablation Study

To quantify the contribution of reference scRNA-seq supervision in SPIDER, we perform three ablation experiments on the CID44971 breast cancer dataset. These ablations isolate the effect of transferring single-cell–derived compositional information to the ST encoder. The results are shown in Table S1.

In the first experiment, we remove the reference scRNA-seq data entirely from the framework, eliminating the branch that injects cell-type compositional information into the model. In this setting, the encoder operating on the real-ST transcriptional graph receives no supervision, and the model learns only from the reconstruction loss in an unsupervised manner. This modification degrades clustering performance (NMI 0.6399, ARI 0.5273, HS 0.6477) relative to the full SPIDER framework (NMI 0.6981, ARI 0.6283, HS 0.6757). These results demonstrate that scRNA-seq supervision contributes meaningfully to modeling the real-ST latent space and ultimately improves denoising.

Model Variant	NMI	ARI	HS
SPIDER	**0.6981**	**0.6283**	**0.6757**
No supervision	0.6399	0.5273	0.6477
Random pseudo-ST supervision	0.5792	0.4266	0.5809

Table S1: **Ablation study demonstrating the contribution of reference scRNA-seq via pseudo-ST data in SPIDER.** Removing the pseudo-ST branch (no scRNA-seq supervision) reduces clustering accuracy across all metrics. Replacing the pseudo-ST data with a random matrix further degrades performance, showing that biologically meaningful pseudo-ST supervision is crucial for learning a coherent ST latent space.

In the second ablation study, we replace the pseudo-ST expression matrix generated from real scRNA-seq data with a randomly generated matrix of the same dimensions but lacking any biological signal. In this configuration, the model still receives "cell-type compositional supervision," but the supervision is uninformative. As a result, clustering performance degrades even further (NMI 0.5792, ARI 0.4266, HS 0.5809). This indicates that the model performs better with no cell-type supervision at all than with misleading supervision derived from random pseudo-ST data. This outcome is expected: without supervision, the embeddings are shaped solely by the reconstruction loss, whereas injecting random supervision distorts the latent space and pulls it away from biologically meaningful structure.

In the third ablation study, we assess the impact of pseudo-ST data generated using different random seeds from the same scRNA-seq reference on real-ST denoising performance. We perform DLPFC denoising using 10 random seeds for pseudo-ST generation. The variability across runs is low, with standard deviations of 0.002 for both NMI and HS and 0.013 for ARI, indicating that the pseudo-ST generation process is robust and yields consistent denoising performance.

The ablation studies demonstrate that the performance of SPIDER critically depends on biologically grounded pseudo-ST supervision. Removing this supervision, or replacing it with random noise, leads to a substantial decline in clustering accuracy, indicating that informative single-cell–derived compositional signals are essential for guiding SPIDER to learn biologically faithful and robust denoised representations of ST data.

S3 Runtime Analysis

We evaluated the computational efficiency of SPIDER and competing methods on two representative ST datasets under identical experimental settings. All experiments were conducted on a workstation equipped with an NVIDIA RTX A4500 GPU. For each dataset, we fixed the number of highly variable genes to 3,000 and trained all models for 500 epochs.

Table S2 reports the total wall-clock runtime for each method on the two datasets. Due to its multi-graph construction, pseudo spot integration, and domain-alignment components, SPIDER requires additional computational cost.

Despite the increased runtime, SPIDER remains practical for typical ST datasets and provides substantial gains in denoising quality and biological interpretability. The observed runtime increase reflects the added modeling complexity necessary to integrate spatial structure with single-cell–derived supervision, rather than inefficient implementation.

Table S2: Runtime comparison of denoising methods. All models were trained for 500 epochs using 3,000 genes on an NVIDIA RTX A4500 GPU.

Dataset	Spots	Method	Runtime (s)
CID44971	1,159	DUSTED	13
		STAGATE	3
		SEDR	13
		stGRL	18
		DenoiseST	14
		SPIDER	21
DLPFC	3,611	DUSTED	42
		STAGATE	5
		SEDR	17
		stGRL	51
		DenoiseST	42
		SPIDER	147

References

[1] Leland McInnes, John Healy, and James Melville. Umap: Uniform manifold approximation and projection for dimension reduction. *arXiv preprint arXiv:1802.03426*, 2018.

[2] F Alexander Wolf, Fiona K Hamey, Mireya Plass, Jordi Solana, Joakim S Dahlin, Berthold Göttgens, Nikolaus Rajewsky, Lukas Simon, and Fabian J Theis. PAGA: graph abstraction reconciles clustering with trajectory inference through a topology preserving map of single cells. *Genome biology*, 20(1):59, 2019.
